# A Novel Role for the Interleukin-1 Receptor Axis in Resistance to Anti-EGFR Therapy

**DOI:** 10.3390/cancers10100355

**Published:** 2018-09-26

**Authors:** Valerio Gelfo, Martina Mazzeschi, Giada Grilli, Moshit Lindzen, Spartaco Santi, Gabriele D’Uva, Balázs Győrffy, Andrea Ardizzoni, Yosef Yarden, Mattia Lauriola

**Affiliations:** 1 Department of Experimental, Diagnostic and Specialty Medicine (DIMES), University of Bologna, 40138 Bologna, Italy; valerio.gelfo2@unibo.it (V.G.); martina.mazzeschi2@unibo.it (M.M.); giada.grilli@studio.unibo.it (G.G.); andrea.ardizzoni2@unibo.it (A.A.); 2 Centre for Applied Biomedical Research (CRBA), Bologna University Hospital Authority St. Orsola-Malpighi Polyclinic, 40138 Bologna, Italy; 3 Department of Biological Regulation, Weizmann Institute of Science, 7610001 Rehovot, Israel; moshit.lindzen@weizmann.ac.il (M.L.); yosef.yarden@weizmann.ac.il (Y.Y.); 4 Institute of Molecular Genetics, National Research Council of Italy, 40136 Bologna, Italy; spartaco.santi@cnr.it; 5 IRCCS-Istitute Orthopaedic Rizzoli, 40136 Bologna, Italy; 6 Scientific and Technology Pole, IRCCS MultiMedica, 20138 Milan, Italy; duva.gabriele@gmail.com; 7 MTA TTK Lendület Cancer Biomarker Research Group, Institute of Enzymology, Magyar Tudósok körútja 2, 1117 Budapest, Hungary; gyorffy.balazs@yahoo.com; 8 Semmelweis University 2nd Dept. of Pediatrics, Tűzoltó utca 7–9, 1094 Budapest, Hungary

**Keywords:** cetuximab, colon cancer, inflammation, colonspheres, MAPK, consensus molecular subtype, resistance

## Abstract

Cetuximab (CTX) is a monoclonal antibody targeting the epidermal growth factor receptor (EGFR), commonly used to treat patients with metastatic colorectal cancer (mCRC). Unfortunately, objective remissions occur only in a minority of patients and are of short duration, with a population of cells surviving the treatment and eventually enabling CTX resistance. Our previous study on CRC xenopatients associated poor response to CTX with increased abundance of a set of pro-inflammatory cytokines, including the interleukins IL-1A, IL-1B and IL-8. Stemming from these observations, our current work aimed to assess the role of IL-1 pathway activity in CTX resistance. We employed a recombinant decoy TRAP IL-1, a soluble protein combining the human immunoglobulin Fc portion linked to the extracellular region of the IL-1-receptor (IL-1R1), able to sequester IL-1 directly from the medium. We generated stable clones expressing and secreting a functional TRAP IL-1 into the culture medium. Our results show that IL-1R1 inhibition leads to a decreased cell proliferation and a dampened MAPK and AKT axes. Moreover, CRC patients not responding to CTX blockage displayed higher levels of IL-1R1 than responsive subjects, and abundant IL-1R1 is predictive of survival in patient datasets specifically for the consensus molecular subtype 1 (CMS1). We conclude that IL-1R1 abundance may represent a therapeutic marker for patients who become refractory to monoclonal antibody therapy, while inhibition of IL-1R1 by TRAP IL-1 may offer a novel therapeutic strategy.

## 1. Introduction

Membrane receptors combine a multitude of extracellular signals like growth factors into multiple signaling pathways and cellular responses. Receptor tyrosine kinases (RTKs) are a subgroup of transmembrane proteins with a tyrosine kinase activity, determining cellular functions such as growth, differentiation, cell motility and survival [1]. The epidermal growth factor receptor (EGFR) is a member of the RTKs superfamily and was the first cell surface signaling protein to be characterized by molecular genetic methods. The EGFR family has been identified as a critical pathway in the signaling from G protein-coupled receptors (GPCRs) [2], cytokines, and integrins, [3] integrating them into an array of cellular responses such as mitogen-activated protein (MAP) kinase activation [4], gene transcription and proliferation. GPCRs are also the targets of key inflammatory mediators, providing a probable link between chronic inflammation and cancer [5]. Indeed, while individual cytokines and receptors have been studied extensively, relatively little is known about the intracellular processing of antagonistic signals. For example, the receptor-interacting protein 1 (RIP1), was shown to regulate the expression of the epidermal growth factor receptor, both in inflammation and stress response [6]. In this vein, we recently unveiled an antagonistic interaction between the EGFR pathway and the steroid receptor for glucocorticoids [7,8], indicating a circadian regulation on the EGFR pathway. Similarly, glucocorticoids have been reported to reduce the production of several inflammatory cytokines [9], such as tumor necrosis factor alpha (TNFA) and interleukin 1 (IL-1), which display a diurnal fluctuation in healthy subjects [10].

The EGFR pathway represents the major target in colorectal cancer (CRC) patients without mutations in *KRAS*, *BRAF* and *NRAS* genes [11]. Indeed, EGFR-directed monoclonal antibodies, such as cetuximab and panitumumab, have been shown to be a valuable treatment option in patients with advanced all-RAS-Wild Type CRC. Unfortunately, only a minority of patients achieve an objective response to this class of agents and duration of tumor regression, when present, is usually limited to the inevitable occurrence of drug resistance and therapy failure [12,13]. Several investigators have proved this profile is the result of cancer heterogeneity, which predicts the overgrowth of resistant clones during the treatment [14,15]. Alternative models, such as pathway bypass or signaling reactivation and microenvironment-mediated cellular changes, have also been implicated in therapeutic failure [16,17,18,19]. Thus far, overcoming resistance to the monoclonal antibody targeting EGFR has represented a major challenge in oncology.

The present work focused on microenvironment-secreted factors, such as cytokines, which are able to protect tumor cells from death and support metastatic spread [20]. Several reports support this model; for example, in lung cancer prompt NF-κB activation upon EGFR inhibition is responsible for tumor cell survival and treatment failure [21]. Furthermore, a factor in the conditioned medium secreted by cells treated with an EGFR inhibitor (erlotinib), proved to confer resistance in otherwise sensitive cell lines [20]. These soluble factors could have several origins, from cancer-associated fibroblasts to tumor-associated macrophages or immune cells resulting from an autocrine/autonomous feedback loop driven by cancer cells [22]. One of the major contributors responsible for the signal transduction between tumor microenvironment and cancer cells is IL-1. IL-1 is a pleiotropic cytokine with several roles in both physiological and pathological states. The IL-1 family of cytokines includes two agonists, IL1A and IL1B, and a specific receptor antagonist, IL-1Ra. IL-1A and IL-1B are agonistic ligands for the IL-1 receptor (IL-1R1) and induce similar biological activities [23]. IL-1 has been implicated in the expression of metastatic, angiogenic and growth factors in many solid tumors [24]. Nevertheless, the role of this pathway in resistance to EGFR targeting monoclonal antibody is still far from being studied.

In this vein, we previously reported that activation of a module of inflammatory cytokines, including *IL1A*, *IL1B* and *IL8*, was associated with impaired therapeutic efficacy in a cohort of xenopatients treated with cetuximab (CTX) [25,26,27]. The current work investigated a possible causative role of secreted IL-1 cytokines in impairing response to CTX. Indeed, patients’ data associated higher IL-1R1 expression levels with poor CTX response. To evaluate the role of the IL-1R1 axis in CTX resistance, we tested the ability of a recombinant decoy, TRAP IL-1, to bind and neutralize IL-1, thereby affecting cell growth, both as monolayers and as colonspheres. We conclude that paracrine/autocrine IL-1 cytokine production plays a role in coordinating cellular response to CTX. Mechanistically, we show that IL-1 pathway activation is responsible for sustaining the AKT and ERK signaling, prevented by TRAP IL-1. Finally, we suggest that IL-1R1 overexpression in poor prognosis CRC patients may represent a valid tool for selecting CTX-sensitive patients.

## 2. Results

### 2.1. Overexpression of IL-1R1 Correlates with Reduced Patient Sensitivity to Cetuximab

**Figure 1:**
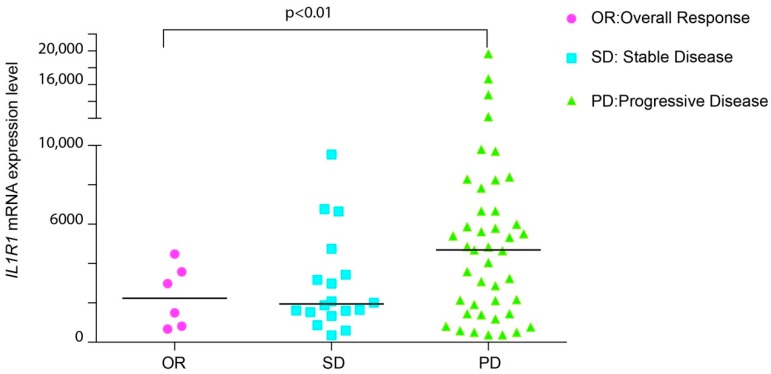
**IL-1R1 abundance predicts response to CTX in patients**. Expression analysis of IL-1 receptor in 67 CRC patients treated with CTX. Responsive patients (OR) as well as stable disease (SD) display low amounts of IL-1R1 compared to the non-responsive patients (PD). The differences in IL-1R1 expression between individual groups (OR/SD/PD) were determined by one-way analysis of variance (ANOVA), followed by Fisher LSD test.

Several clinical studies indicate that overexpression of inflammatory cytokines, such as IL-1, IL8, IL6 or CXCL1, correlates with cancer progression and decreased response to EGFR targeting therapy [25,27]. We previously reported that the abundance of IL-1 cytokines predicts sensitivity to EGFR blockage [26]. To further assess the clinical relevance of our results, we explored whether the receptor for IL-1, namely IL-1R1, was enriched in tumors from patients exhibiting attenuated response to anti-EGFR antibodies. We started by mining the gene expression profile from a publicly available microarray data set, comprising KRAS wild-type CRC patients, treated with cetuximab monotherapy, as described by Khambata-Ford et al. [28] (GSE5851). Only one sample was excluded, because of its uncertain rectal origin. We observed that IL-1R1 is overexpressed in a distinct subpopulation of patients categorized as progressive disease (PD), defined as an increase in tumor burden upon CTX treatment. Consistently, patients with stable disease (SD) or an overall response (OR) to CTX therapy displayed lower IL-1R1 expression than the non-responding patients (Figure 1). In line with our previous work [26], we conclude that increased expression of both IL-1 ligands (IL-1A and IL-1B) and receptor (IL-1R1) is associated with resistance to EGFR targeted therapy.

Next, we evaluated the association of IL-1R1 with AREG and EREG, two EGFR ligands whose expression was previously reported to determine CTX efficacy. Indeed, high levels of AREG and EREG are predictive of response to CTX [28], whereas high levels of IL-1R1 are predictive of poor response (Figure 1). Pearson analysis reported a moderate negative correlation between IL-1R1, AREG and EREG (−0.5, *p* = 0.00057 and −0.44 *p* = 0.003308 respectively, Table 1) in the progressive disease subgroup. These data support the notion that IL-1R1 is a marker of decreased patient sensitivity to CTX blockage, pointing to a role of this pathway in the progression and aggressiveness of colon cancer.

### 2.2. A Recombinant Decoy Containing IL-1R1 Inhibits Growth In Vitro

To address the question whether intercepting “IL-1” cytokines (which refers both to alpha and beta isoforms in the following text), would improve response to EGFR-targeted therapies, we developed an engineered cell system producing a soluble receptor, named TRAP IL-1, comprising the extracellular domain of IL-1R1 linked to a soluble Fc domain of immunoglobulin G as previously described [29].

**Figure 2:**
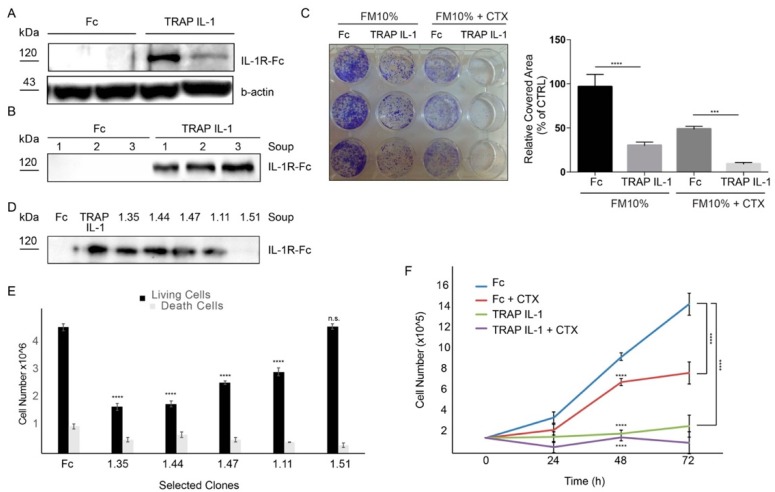
**A recombinant decoy containing IL-1R1 inhibits Caco-2 growth.** (**A**) Western blot analysis of Caco-2 TRAP IL-1 and Fc. Actin served as loading control. 500,000 cells were plated in medium with 10% FBS, then cells were serum starved overnight. The day after cells were harvested and total proteins extracted for TRAP IL-1 detection. (**B**) Western blot analysis of three replicates of Caco-2 Fc and Caco-2 TRAP IL-1 soup. 500,000 cells were plated in DMEM supplemented with 10% FBS and after 5 days the soup was harvested and 20 μL used for the analysis. (**C**) Colony-forming assay of Caco-2 Fc and Caco-2 TRAP IL-1. 4000 cells/well were plated and grown in the absence or presence of CTX (5 μg/mL) for 10 days in medium containing 10% of serum. Cells were then fixed, stained with crystal violet and photographed. Representative figures (left) and quantification (right) of the covered areas by ImageJ are provided. The statistic was calculated by 2-way ANOVA, *** *p* < 0.0005. These experiments were repeated at least three times. (**D**) Western blot analysis of Caco-2 TRAP IL-1 clones soup. 1.35–1.44–1.47–1.11 and 1.51 are clones derived from a single cell. Each clone soup was collected 5 days after seeding. TRAP IL-1 (purified protein) and Fc are intended as positive and negative controls respectively. (**E**) Clones from D were seeded and both living and death cells were counted. Statistical analysis was performed by one-way ANOVA, comparing the mean of proliferation of each clone to the control cells. Dunnet correction for multiple comparisons was applied. **** *p* < 0.0001. (**F**) Cell count of Caco-2 Fc and Caco-2 TRAP IL-1 (clone 1.35). 100,000 cells/Petri were seeded with 10% of serum. After 24 h medium was changed with 10% of serum in the presence or absence of CTX (5 μg/mL) and cells were counted after 24, 48 and 72 h. A 2-way ANOVA was performed, by comparing the matched values for each time point (24, 48 and 72 h) to the Fc control cells. **** *p* < 0.0001.

Our IL-1 inhibition strategy assumes that the ligand-binding specificity of IL-1R1 would be able to sequester the majority of IL-1 ligands, thereby intercepting essential autocrine/paracrine loops. As an initial step, we designed a construct of the IL-1R1 binding domain fused to a six-histidine tag. Then, we stably transduced this construct in CRC Caco-2 cells (TRAP IL-1 cells). By Elisa assay, we confirmed that TRAP IL-1 was able to bind specifically the cognate ligands IL-1A and IL-1B (Supplementary Figure S1). Analyses of total cell lysate showed the successful stable integration of TRAP IL-1 (Figure 2A), which was secreted in the growing medium of TRAP IL-1 stable clones, whereas the Caco-2 control cells (Fc) displayed no product (Figure 2B). Importantly, Fc cells displayed undisturbed proliferation and partially responded to CTX treatment, while TRAP IL-1 cells displayed impaired proliferation and enhanced ability to respond to CTX treatment (Figure 2C). Next, we tested several colonies for the amount of secreted TRAP IL-1 in the medium. In detail, medium was collected after five days in culture and analyzed by western blot. Purified TRAP IL-1 (diluted at 10 μg) was used as a positive control (Figure 2D). The proliferation capability and cell death in TRAP IL-1 clones was then evaluated and reported (Figure 2E). Interestingly, TRAP IL-1 positive clones displayed decreased proliferation (Figure 2E), without significant changes in cell death (Figure 2E), thereby excluding a toxic effect of the secreted TRAP IL-1. We then employed one of the clones characterized by high TRAP IL-1 production for further phenotypical evaluations, confirming that cells stably overexpressing the TRAP IL-1 display impaired growth compared to the Fc control (Figure 2F). These in vitro data support TRAP IL-1 adjuvant role in inferring with cell proliferation and CTX response.

### 2.3. TRAP IL-1 Clones Display Decreased Cancer Cell Spheroidogenesis in 3D

**Figure 3:**
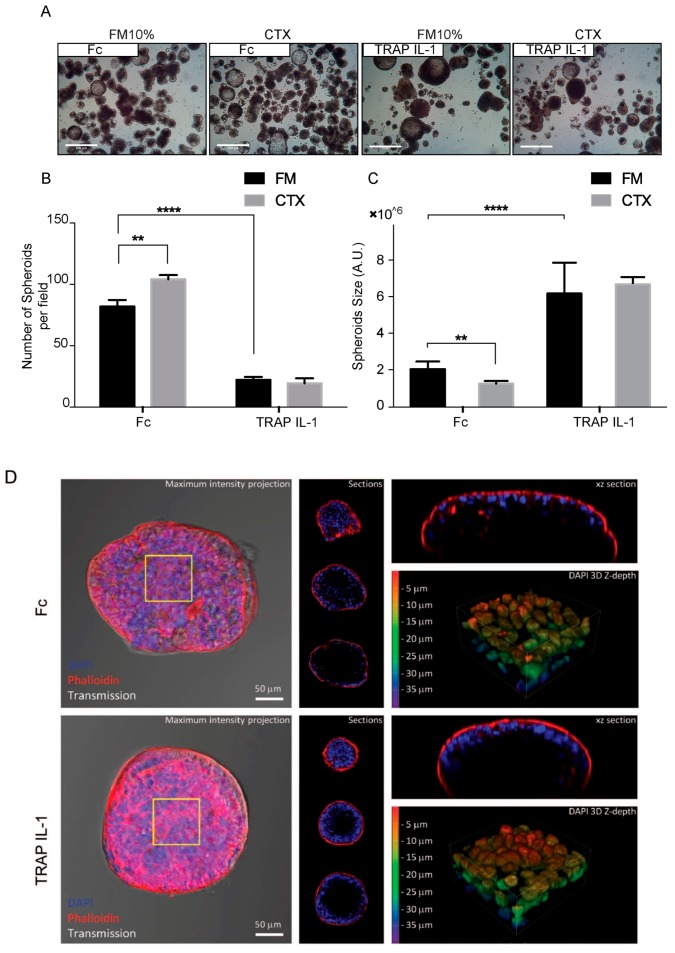
**Caco-2 TRAP IL-1 display decreased growth in suspension as colonspheres.** (**A**) 4× magnification of Caco-2 Fc and Caco-2 TRAP IL-1 producing “spheroid-like” structures, under the indicated treatments. Scale bar 100 μm. (**B**) Number of filled spheroids presented as average ± S.E.M. 2-way ANOVA with Bonferroni Test, ** *p* < 0.01; **** *p* <0.0001; (**C**) Quantification of spheroid size measurements under the indicated treatments in 10% FBS medium supplemented with EGF 10 ng/mL and CTX 5 μg/mL. Columns represent volume averages ± S.E.M, 1-way ANOVA, ** *p* < 0.01; **** *p* < 0.0001. (**D**) 3D confocal microscopy of colonspheres Fc and TRAP IL-1. In the left panel is reported the DAPI and phalloidin staining. In the middle panel are featured single optical sections collected at 15 μm intervals. In the panel on the right, the xz optical section passing through the maximum diameter of the spheroids (upper part) and 3D rendering focused on the nuclear density and z-depth of the spheroids (yellow square), reported as a scale of colors, red indicates a 5 μm depth and blue 35 μm depth (bottom part).

We sought to identify the phenotype of TRAP IL-1 in a defined 3D microenvironment, based on the lack of attachment to the plastic tray and forcing the cells to grow as spheroids. Fifteen days after suspending single cells in EGF supplemented medium, Fc cells formed hollow lumen cysts (Figure 3A). Similarly, TRAP IL-1 expressing cells were also able to form spheroids cyst-like structures, but these appeared rounded and smoother than the Fc counterpart (Figure 3A). Next, we evaluated the Fc derivative both for colonsphere size and number. Fc appeared smaller than TRAP IL-1, which on the counterpart retained a decreased ability to form spheres (Figure 3B). Indeed, by measuring more than 200 spheroids per condition, we found that the average size of TRAP IL-1 was larger than Fc, but the number of spheres was significantly lower (Figure 3C).

By confocal microscopy, we analyzed the inner organization of Fc and TRAP IL-1 spheres. Fc spheres appeared loose and with low nuclear density on the outermost layer, while TRAP IL-1 displayed a pronounced nuclear density and an overall organization, suggesting an increased cell polarity. Fc displayed enhanced actin accumulation at the internal surfaces lining the central cavity, with a clear lumen, which was present in the majority of the cells.

These results are in line with previous reports by time-lapse microscopy suggesting that the hollow lumen occurs through a process of internal expansion without cell proliferation or cell death [30,31]. On the other hand, TRAP IL-1 still retained a wide and hollow lumen, but the cyst-like formation was much more emphasized in comparison to the control, while the ability of forming spheres was dramatically decreased in these cells (Figure 3B–D). These data suggest a role for the TRAP IL-1 decoy in impacting on cells growth both in monolayers and in 3D.

### 2.4. IL-1 Pathway Inhibition Impairs MAPK Signaling

**Figure 4:**
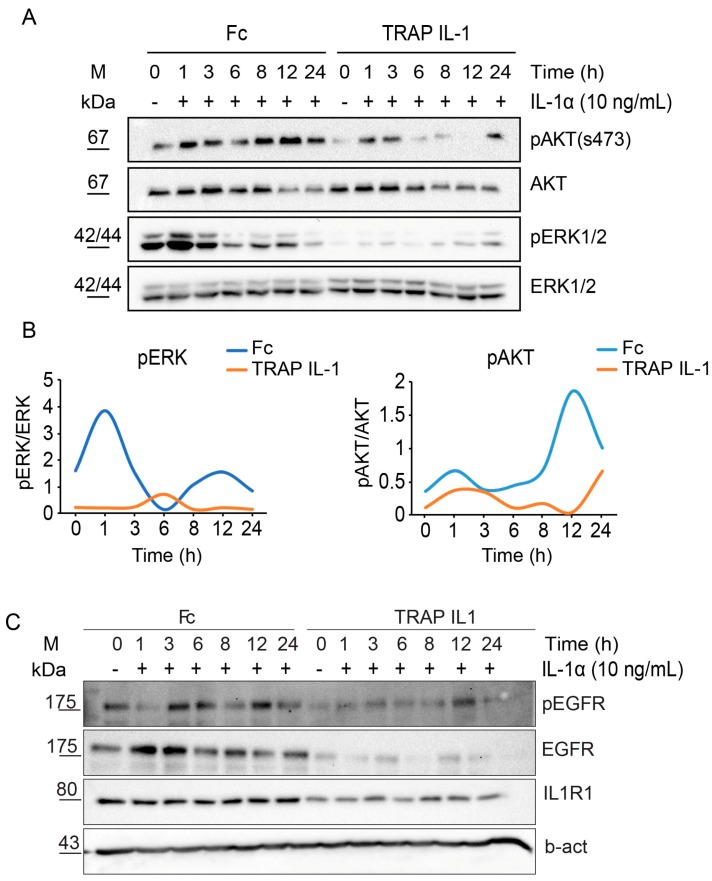
**Bimodal activation of ERK and AKT after stimulation with IL-1A.** (**A**) Western blot analysis of phospho-AKT s473 (pAKT) and phospho-ERK (pERK) levels in Caco-2 Fc and Caco-2 TRAP IL-1 cells (time course). At day one, 500 k cells were plated for each condition in medium with 10% FBS. At day 2, cells were serum starved overnight and the day after IL-1A (10 ng/mL) was added to the medium of growing cells for 1, 3, 6, 8, 12 and 24 h. After treatment cells were harvested, total proteins extracted and quantified. Monoclonal antibody against total AKT and ERK1/2 served as loading control. (**B**) Quantification of pAKT and pERK by Image Lab is provided. (**C**) Western blot analysis of phospho-EGFR Tyr1068, EGFR and IL-1R1 in Fc and TRAP IL-1 cells treated as in A. B-actin served as loading control.

A functional secreted and soluble TRAP IL-1 should be able to neutralize IL-1 ligands. Thus, we treated Fc and TRAP IL-1 cells with IL-1A (10 ng/mL) over a long-time course, up to 24 h. To assess the effect of IL-1 activity, we analyzed both the mitogen activated protein kinase (MAPK) and the AKT axes. IL-1A treatment displayed a signaling activation characterized by a specific pattern, with an immediate mild activation of both ERK and AKT signals, followed by a secondary and delayed activation, as reported in the quantification in Figure 4B. According to these results, TRAP IL-1 was able to nullify the action of IL-1 on MAPK signaling activation, thus proving an effective inhibition of IL-1 stimulus in vitro. Furthermore, TRAP IL-1 production spared the basally active ERK and AKT. Interestingly, both ERK and AKT displayed a bimodal activation (Figure 4B), which was also blunted by TRAP IL-1. Next, we tested the abundance and the activation of EGFR under IL-1A stimulus, by measuring the phosphorylation of tyr1068. We detected and increase in EGFR abundance upon 3 h of IL-1A treatment, with a consistent increase of phosphorylated EGFR. This effect could be related to an impaired degradation followed by a fast recycling of the receptor to the cell surface, a phenomena well described under TNFA or UV stress [32]. Secondarily, we detected a new hit of phospho-EGFR at 12 h of treatment, which was not followed by an enhanced EGFR protein, and it was consistent with the bimodal activation of MAPK and AKT. These data suggest that IL-1 boosts EGFR levels and that a positive feedback loop engaged by IL-1R1 stimulation may be responsible for sustaining the MAPK and AKT signals, through secondary activation of EGFR pathway. TRAP IL-1 was able to blunt EGFR production with a very low amount of pEGFR, an effect attributable to a decreased EGFR abundance, which might explain an overall lower activation of the downstream signaling pathways (Figure 4A,C). Moreover, TRAP IL-1 is influencing endogenous expression of IL-1R1, which is stable in control cells under IL-1 treatment, but it appears downregulated in TRAP IL-1 cells. We speculate that this is the consequence of a chronic sequestration of IL-1 that might be responsible for a loss of dependency from this receptor in our cell system (Figure 4C). To sum up, these results indicated that secreted TRAP IL-1 molecules successfully dampen EGFR abundance and lead to a decreased activation of ERK and AKT.

### 2.5. IL-1 Receptor Abundance Predicts Relapse-Free Survival in CRC Patients

IL-1A and IL-1B signal through the same receptor complex. The response is initiated when the ligand binds to its primary receptor subunit IL-1R1 [33]. The receptor contains extracellular immunoglobulin domains and a Toll/IL-1 receptor domain in the cytoplasmic portion. Binding of the ligand allows the recruitment of a second receptor subunit, the IL-1R1 accessory protein (IL-1RAP). Formation of the receptor heterodimer induces signaling because the juxtaposition of the two Toll/IL-1 receptor domains enables the recruitment of myeloid differentiation primary response protein 88 (MYD88), IL-1R1 associated kinase 4 (IRAK4), tumor necrosis factor receptor-associated factor 6 and other signaling intermediates [34]. In vitro, our data pointed to an involvement of IL-1 in tumor growth and the lack of response to EGFR interception. We further addressed the question of IL-1R1 expression associated with CRC progression in patients by performing a bioinformatic study in a cohort of 2166 CRC patients [35]. The Kaplan-Meier estimated the fraction of CRC patients resected for colorectal cancer and having a follow-up of a period of 200 months. We compared the relapse free survival (RFS) of 1211 patients, by splitting the dataset according to each cut off level between the lower and upper quartile of the expression level of the IL-1R1 gene. We computed false discovery rate (FDR) to correct for multiple testing, and accepted only results with a FDR below 5%. The initial analysis was not restricted to specific categories, and interrogated the entire dataset. We distinguished two sets of patients with high and low IL-1R1 mRNA (probe 202948_at) expression respectively, and the association with patient survival. High levels of IL-1R1 were predictive of a worse disease-free survival and death with an HR of 1.75 and *p*-value of 1.6 × 10^−6^. Median relapse-free survival was 21 months and 66 months in the low and high IL-1R1 expression cohort, respectively.

**Figure 5:**
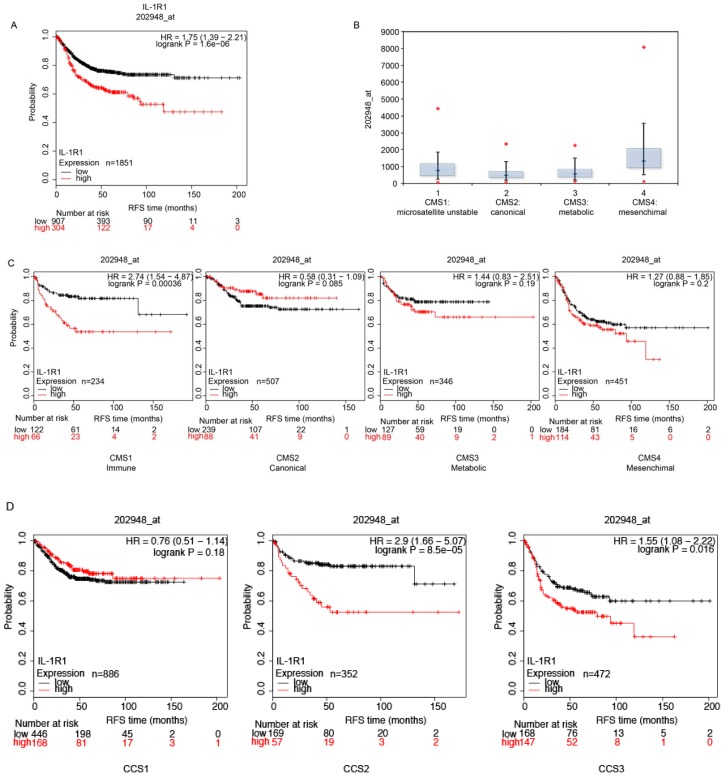
**IL-1 receptor expression predicts survival in CRC patients.** (**A**) A cohort of 1211 patients was divided into two groups according to IL-1 receptor abundance. In the Kaplan-Meier plot, the black line represents patients with an overall low IL-1R1 expression, while the red line represents subjects with high expression of IL-1R1. For each patient, the relapse free survival (RFS) is reported over time and expressed in months. (**B**) mRNA abundance of IL-1R1 in the four consensus molecular subtypes (CMSs). (**C**) Stratification of patients using the CMS criterion. Patients are divided into four subtypes. CMS1 (Immune): hypermutated, microsatellite unstable and strong immune activation. CMS2 (Canonical): epithelial, marked WNT and MYC signaling activation. CMS3 (Metabolic): epithelial and evident metabolic dysregulation. CMS4 (Mesenchymal): prominent transforming growth factor-b activation, stromal invasion and angiogenesis. (**D**) KM plot of IL-1 receptor expression using the de Sousa Classification of Colon Cancer subtypes.

These results highlight the role of IL-1R1 in CRC patients and suggest that IL-1R1 interception could represent an effective clinical strategy to improve prognosis and survival. Next, we attempted to stratify colorectal tumors into unique features according to their genetic profiles. We employed the consensus molecular subtype (CMS) as classified by Guinney et al. [36]. They showed a marked interconnectivity among six independent classification systems coalescing into four CMSs with distinguishing features; CMS1 (microsatellite instability-immune), covering about 14% of CRC tumors, CMS2 encompassing the canonical subtype with epithelial markers, CMS3 characterized by a metabolic dysregulation, and CMS4 with TGFβ activation, stromal invasion and angiogenesis. The abundance of IL-1R1 appears higher in CMS4 and CMS1, than in CMS2 and CMS3 (Figure 5B). IL-1R1 had a strong impact on patient survival in the CMS1 subtype (Figure 5C), with an impressive HR of 2.74 and *p*-value: 0.00036.

We then employed the de Sousa stratification, which associates patients with three distinguished colon cancer subtypes (CCS) CCS1, CCS2 and CCS3. CCS1 patients have the lowest risk of recurrence after tumor resection compared to patients belonging to the other subtypes [37]. Interestingly, IL-1R1 overexpression is associated with more aggressive subtypes (Figure 5D), with a strong association in CCS2, HR of 2.9 and *p*-value: 8.5 × 10^−5^ and in CCS3, HR of 1.55 *p*-value: 0.016 but in this case FDR was over 50%, whereas no association was found for CCS1. Notably, IL-1R1 overexpression is also predictive of survival in the CCS3 subtype, which has very unfavorable prognosis and is refractory to EGFR-targeted therapy.

## 3. Discussion

The machinery of the ErbB pathway is conserved throughout evolution, and has been studied as a linear pathway for decades. Binding of the ligand to the monomeric receptor promotes receptor dimerization and self-phosphorylation on tyrosine residues in the catalytic domain. In higher eukaryotes, this linear pathway has evolved into a richly interactive, multilayered network conferring selective gains in terms of adaptation, tolerance to mutations and signal diversification [38].

Our model assumes that resistance to EGFR blockage is mediated by the upcoming activation of an alternative pathway, namely the IL-1R1. These findings are fundamental for effective clinical intervention, indeed immunotherapy stimulating anti-tumor immunity represents an increasingly successful pillar in cancer care [39] and it is opening the way to novel therapeutic strategies that could be combined with available immunotherapeutic regimes.

The inflammatory microenvironment is becoming a score to predict immunotherapy response, with a strong immune engagement predicting either a good or poor cancer outcome [40,41]. For example, high levels of lymphocytes are predictive of a better outcome [42], whereas a low lymphocyte-to-monocyte ratio is predictive of a worse survival in CRC patients [43]. The general consensus is that high levels of monocytes are associated with tumor progression and metastasis [44]. Intriguingly, monocytes/macrophages represent the main source of IL-1 production in the tumor microenvironment [33,45]. Although their role in modulating the efficacy of oncogene-targeted therapy has yet to be elucidated, there is increasing evidence that tumor-associated macrophages contribute to the cytotoxicity of therapeutic monoclonal antibodies (moAbs) [46]. For example, CTX resistant patients show a baseline increased expression of gene signatures specific for monocytic infiltrates [47], while macrophage engagement by the serum of anti-EGFR cetuximab was shown to enhance the immunosuppressive, proangiogenic and protumoral functions of tumor-associated macrophages both in animals [48] and humans [49].

Our results therefore suggest that microenvironment-derived IL-1 may impact on CTX action in a subset of CRC patients featuring IL-1R1 overexpression. IL-1 activation is associated with several cancer types and the expression of this cytokine increases during CRC progression [50,51,52,53]. We previously reported that in vitro CTX treatment is responsible for IL-1 production [26]. Indeed, the subset of patients with progressive CRC presented higher levels of IL-1R1 than patients responsive to the therapy or at least with a stable disease. Consistently, we found an impressive association of IL-1R1 to CRC outcome. But, interestingly, this was specifically predictive for CMS1, also known as the immunological subtype, mainly characterized by microsatellite instability [54]. Of note, CMS1 is characterized by increased expression of genes associated with a diffuse immune infiltrate, along with strong activation of immune evasion pathways [55].

Mechanistically, we showed that IL-1R1 activation by IL-1A stimulus is responsible for MAPK engagement and AKT activation. We assume that in this context, CTX activity will be dampened by the secondary loop of positive feedback activation responsible for sustaining of ERK and AKT activation. The delayed kinetic of EGFR activation suggests a preceding cascade of cellular events ignited by IL-1 treatment. Our model suggests a transregulatory mechanism mediated by IL-1 pathway activation that entails EGFR receptor phosphorylation in trans and evasion from the degradative fate. These data are in line with a model of recycling of EGFR consequent to cytokine treatment, as well as UV and tumor necrosis factor, as previously described [32]. Furthermore, it may be closely linked to another important positive late feedback mechanism given by the autocrine loop, mediated by the MAPK pathway, that sustains growth factors signals such as tumor necrosis factor, interleukin-1A and IL-1 receptor antagonist, and converts a transient stimulus into a sustained signal [56,57].

In addition, our in vitro data show that inhibition of IL-1R1 signaling by sequestering the IL-1 ligands from the microenvironment may represent a successful strategy to enhance response to therapy [23,58]. Under chronic IL-1 neutralization, the decreased number of spheroids formed by cancer cells, along with the empty cyst-like shape may represent a systemic change towards a differentiated profile. We predict that when injected subcutaneously into athymic nude mice, the TRAP IL-1 cells will form well differentiated, encapsulated tumors, similarly to the phenotype previously reported [59], whereas Fc cells will form poorly differentiated and locally invasive tumors [59], although further studies in vivo will be required to prove these hypothesis.

## 4. Materials and Methods

### 4.1. Cell Culture

The experiments were conducted on the Caco-2 TRAP IL-1 cells, which are a colorectal adenocarcinoma cell line stably transfected with TRAP IL-1 plasmid, along with Caco-2 Fc cells transfected with a plasmid control. Engineered cells were maintained in Dulbecco’s minimal essential medium (DMEM), supplemented with 10% of fetal bovine serum (FBS) and antibiotics (1% penicillin-streptomycin and 1% amphotericin B) in a 37 °C atmosphere containing 5% CO_2_. Caco-2 cells were authenticated by short tandem repeat (STR) profiling (PowerPlex 21 PCR Kit, Promega, Madison, WI, USA) and the certificate was released by the external service Eurofins Medigenomix Forensik GmbH (Ebersberg, Germany). Cells were routinely tested for mycoplasma contamination.

### 4.2. Colony Forming Assay

The colony-forming assay was used to compare the degree of transformation of engineered Fc and TRAP IL-1 cells according to their ability to grow at a reduced density. Two thousand cells were seeded in 12-well plates with and without CTX (5 μg/mL). Treatments were added immediately, as reported in the figure legends. After ten days, the medium was removed, and the cells were washed with PBS and fixed with a solution of methanol and acetone (1:1) for 20 min at −20 °C. After washing with PBS, the cells were stained with crystal violet 0.05% for 30 min, then washed with water to remove any excess of dye. A picture of each well was taken and the covered area was measured using ImageJ software. For each treatment, the mean value of the covered area was calculated as a percentage of the control.

### 4.3. Spheroid Assay

To assess the ability of cells to grow in anchorage-independent conditions, a growth assay in “low-attachment” condition was carried out. Six-well plates were covered with a layer of 0.6% agar. For each well, 10,000 cells were seeded in 2 mL of 10% FBS medium, supplemented with EGF (10 ng/mL). Treatments were added immediately. After two weeks of treatment, pictures of four non-overlapping fields for each well were collected using an inverted microscope (Leitz Fluovert, code 520) at 4× magnification. Spheroids from each condition were counted, and the lengths of the major and minor axes were measured using ImageJ Software. Values below 70 μm were filtered as not corresponding to mature spheroids and the volume was calculated applying the sphere adapted formula (major axis × minor axis^2^)/2.

### 4.4. Confocal Microscopy

We used confocal microscopy to investigate the inner structure of spheroids and potential phenotypical changes in response to drugs treatment. Spheroids from 3D culture were collected, centrifuged (1000× *g* for three minutes) and washed with PBS. Next, spheres were fixed in 4% PFA for 1h, washed and stored in PBS. The spheres were then stained with phalloidin (5 μg/mL; #A12379, Thermo Fisher Scientific, Waltham, MA, USA) and DAPI (1 μg/mL; #D9542, Sigma-Aldrich, St. Louis, MO, USA). The confocal imaging was performed with a confocal laser scanning microscope (Nikon A1-R) equipped with a 20 × 0.7 NA objective and 405 and 561 nm laser lines to excite DAPI and TRITC fluorescence signals, (resolution: 1024 × 1024 pixels; gray levels: 4096). Volume view with 3D rendering was carried out using the NIS Elements Advanced Research software (Nikon, Minato, Tokyo, Japan).

### 4.5. Immunoblotting

Cells were lysed with RIPA buffer supplemented with protease inhibitor cocktail (P8340, Sigma-Aldrich) and phosphatases inhibitors (Na_3_VO_4_) at a final concentration of 1 mM, incubated for 20 min on ice. Protein concentration in the supernatants was determined by DC Protein Assay (Bio-Rad, Hercules, CA, USA), using bovine serum albumin as standard and proteins (50 μg of total lysate) were loaded. The following primary antibodies were used: anti-AKT rabbit polyclonal antibody (100 ng/mL, #9272), anti-phospho-AKT (Ser473) (D9E) XP rabbit monoclonal antibody (100 ng/mL, #4060), anti-EGFR (D38B1) (29 ng/mL, #4267), anti-EGFR phospho tyr1068 (D7A5) (423 ng/mL, #3777) (Cell Signaling Technology, Danvers, MA, USA); anti-ERK2 (D-2) mouse monoclonal antibody (200 ng/mL, sc-1647), anti-IL-1R1 (H-8) mouse monoclonal antibody (400ng/mL, sc-393998), anti-B-actin (400 ng/mL, sc-47778) (Santa Cruz Biotechnology, Dallas, TX, USA) and anti-p44/42 MAPK (Erk1/2) mouse monoclonal antibody (10 ng/mL, M1859, Sigma Aldrich, St. Louis, MO, USA). Peroxidase-conjugated AffiniPure Goat Anti-Human IgG, Fc_γ_ Fragment Specific (800 ng/mL, 109-035-098, Jackson ImmunoResearch, Ely, Cambridgeshire, UK). Protein was detected by incubation with anti-rabbit or -mouse horseradish peroxidase-labeled secondary antibody (Dako EnVision+ System- HRP Labelled Polymer) followed by chemiluminescent reaction (Clarity Western ECL Substrate, Bio-Rad). Chemiluminescence was detected with the ChemiDoc XRS+ system (Bio-Rad).

### 4.6. Statistical Analysis

The statistical analyses were performed by using Prism version 6 (GraphPad Sotfware, Inc., La Jolla, CA, USA). The one way or two-way ANOVA were used to test significance of the assays. * *p* < 0.05, ** *p* < 0.01, ** *p* < 0.005, **** *p* < 0.0001.

## 5. Conclusions

Understanding how autocrine cascades operate in normal and pathological tissues seems likely to yield significant therapeutic insights, particularly with respect to multidrug therapies. Tumors not only manage to escape the host immune system, but effectively contrive to benefit from infiltrating cells by modifying their functions to create the microenvironment favorable to tumor progression.

Lastly, we predict that efficient tumor treatment will target both the malignant cells and the tumor microenvironment. The astonishingly reduction of incident lung cancer cases among patients with prior myocardial infarction treated with the anti-IL-1b antibody (canakinumab) in lung cancer [60] are indeed opening a new scenario for the crucial role of the IL-1 cytokine in cancer development. Thus, it is reasonable to consider either an adjuvant role for canakinumab alone or in combination with CTX, might constitute a future treatment for patients who are refractory to CTX monotherapy. The mechanisms underlying this strategy remain obscure, but this work points to the involvement of MAPK and AKT axes. The combination of the signaling under CTX and anti-IL-1 antibodies awaits future investigation.

## Figures and Tables

**Table 1:** Pearson correlation of IL-1R1 to AREG and EREG.

	AREG vs. IL-1R1, *p*-Value	EREG vs. IL-1R1, *p*-Value
PD	−0.50 (0.00057)	−0.44 (0.003308)
SD	−0.55 (0.01881)	−0.47 (−0.04905)
OR	−0.05	−0.08

PD: Progressive disease, SD: stable disease, OR: Overall Response.

## Supplementary Materials

The following are available online at http://www.mdpi.com/2072-6694/10/10/355/s1. Figure S1: Ligand-binding detection of TRAP IL-1.
