# Supplementary material for: A Novel Role for the Interleukin-1 Receptor Axis in Resistance to Anti-EGFR Therapy
Source: Cancers (Basel). 2018 Sep 26;10(10):355. doi: 10.3390/cancers10100355 (PMC6210663; doi:10.3390/cancers10100355)
Supplement: Supplementary file 1 [file cancers-10-00355-s001.pdf]

Supplementary Materials: A Novel Role for the Interleukin-1 Receptor Axis in Resistance to Anti-EGFR Therapy

Valerio Gelfo, Martina Mazzeschi, Giada Grilli, Moshit Lindzen, Spartaco Santi, Gabriele D'Uva , Balázs Győrffy, Andrea Ardizzoni, Yosef Yarden and Mattia Lauriola

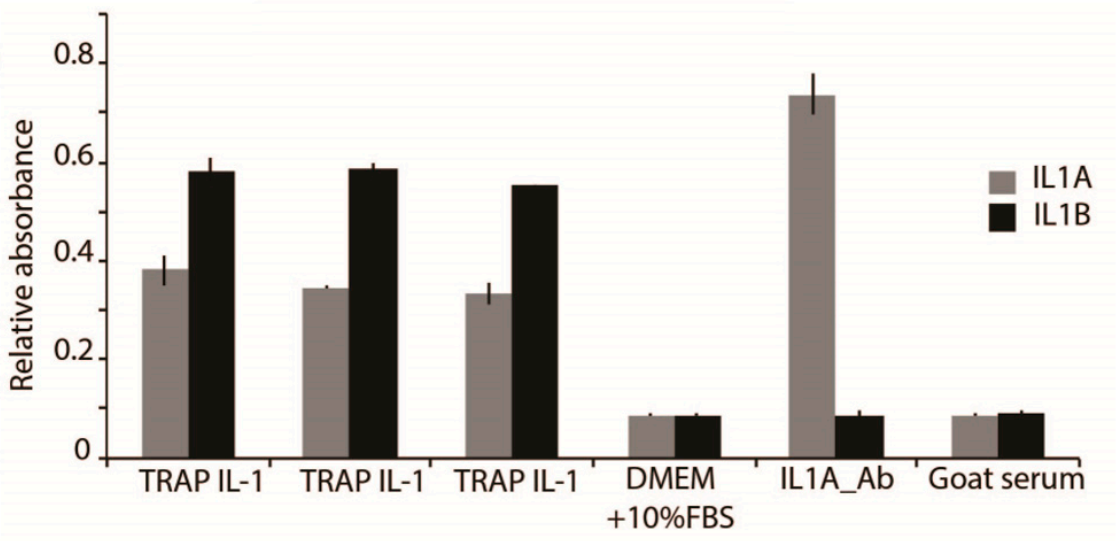

Figure S1. Ligand-binding detection of TRAP IL-1. Elisa assay testing the ability of three TRAP IL-1 clones, here referred to as TRAP 1/2/3, to specifically bind purified IL-1A/B. Briefly, 96 wells plates were coated with IL-1A and IL-1B and TRAP medium was added. After incubation, medium was washed out, and absorbance was detected. DMEM with 10% FBS and Goat serum served as negative control, a commercial anti IL-1A antibody was used as positive control.
